# A Dietary for Mucous Colitis

**Published:** 1909-01-09

**Authors:** 


### Medicine.

### A DIETARY FOR MUCOUS COLITIS.

The term mucous colitis is commonly used to denote an exceedingly troublesome symptom-complex, of which the main features are the passage of lumps or strings of mucus per rectum from time to time, with quiescent intervals; obstinate constipation, relieved only with extreme difficulty, and then sometimes replaced by transient diarrhœa; paroxysms of severe abdominal pain about the same time that the mucus is being passed; and an occasional alvine discharge of intestinal sand—all these things occurring in a patient who is nervous, hysterical, or neurasthenic, and is commonly an unmarried woman between 25 and 50 years of age.

It is important to add to the above brief account of the disease that it is not in itself fatal, and that it is not accompanied by any obvious macroscopic lesion in the intestine. Somewhat similar symptoms may occur in cases of tuberculous or malignant ulceration of the intestine, chronic dysentery, ulcerative colitis, and in entero-colitis such as may result from chronic obstruction to the bowel by bands or peritoneal adhesions. In all of these conditions there may be inflammation of the intestine, and mucus may be passed per rectum either at the same time as the fæces or independently. Time and usage, however, have given the term "mucous colitis" a very special and restricted meaning, and although "mucous colic" would express that meaning better, mucous colitis is so well established as the name of a particular condition that it would be hard to change it now.

Although an inflammation of the bowel may result in some cases of long standing, especially if purgatives have been misused, the condition is not primarily an inflammation. The mucus that passes per rectum has two chief forms: one, a viscid mass like egg albumen, the other a more definite shape, like membrane, or bands, or strings, often suggesting (to the patient) tape worms; but in neither case are there cellular elements, indicative of inflammation, to be seen under the microscope. Although chronic constipation is nearly always a preliminary factor, and although some obvious neurosis is usually an associated condition, the combination of constipation and nervousness does not necessarily produce mucous colitis. Some affection of the nervous apparatus that governs the secretion of the large intestine seems necessary as well; in other words, mucous colitis is due to a secretory neurosis of the bowel. It is a form of hysteria, but a particular form which is very apt to persist.

When there is no doubt that a patient is suffering from mucous colitis in its restricted sense, treatment of the most careful kind becomes necessary. It is our purpose just now to refer to one part of that treatment only—namely, the dietary; but we would just mention two other points in passing—namely, the great amount of harm that may be done in these cases by the ill-judged use of purgatives and the great amount of good that may be accomplished by insisting upon the attempt to have the bowels opened daily at the same time without fail, whether the desire to defæcate exists at that time or not. The restoration of the diurnal function of the bowel by the latter process, slow though it may be, is of far greater value than is any temporary evacuation by drugs which disturb that function.

### The Dietary in Detail.

Turning now to the details of the dietary for a case of mucous colitis, no hard and fast rules can be laid down, owing to the idiosyncrasies of patients; but the general principles upon which Von Noorden lays stress are that the food shall contain a bulky residue, and that the irritating effects of such a diet shall be minimised by adding to it large quantities of fat in various forms. Cellulose is the most important residue in Von Noorden's dietaries for mucous colitis. The following is a practical account of the details of such a *régime*, open to suitable modification according to circumstances, as advocated by Dr. A. C. Ransome.

It is better that the patient should be isolated in a nursing home, with a firm but sympathetic nurse; and the treatment may be the same, whether it is begun during an acute attack of the malady or during an interval. The patient should go to bed and stay there. A typical dietary would be as follows:—

7 a.m. Half a pint of milk-cream mixture.

8 a.m. Half a pint of Kissingen water.

9 a.m. Half a pint of cocoa with cream, 2 ounces of bread, 2 ounces of butter, marmalade.

11.30 a.m. Twelve ounces of special soup, 3 ounces of bread, 1 ounce of butter.

1 p.m. *Lunch*: Pounded meat, 2 ounces of bread, boiled potatoes, green vegetables, baked apple, stewed pears, or boiled gooseberries, and cream.

4 p.m. Half a pint of milk-cream mixture.

7 p.m. *Dinner*: like lunch, but with 3 ounces of bread and 2 ounces of butter.

9.30 p.m. Half a pint of milk-cream mixture.

Dr. Ransome adds some practical notes as to the nature of milk-cream mixture, and as to the mode of preparation of some of the foods. Milk-cream mixture consists of equal parts of milk and cream, and one teaspoonful of sugar of milk per pint. The cream should contain 30 per cent. of butter-fat, and nearly a pint of it should be taken in 24 hours in one form or another. The bread should be of the coarsest flour obtainable. The larger proportion of husk it contains the better. The usual brown bread sold as whole-meal bread is not sufficiently coarse.

Dr. Ransome lays particular stress upon the value of special vegetable soup, and he gives the following directions for its preparation: A breakfast cupful of lentils or dry peas are placed in a pan in sufficient cold water to cover them, and they are allowed to soak all night. In the morning a slice of fat bacon 6 in. by 2 in. by $\frac{1}{4}$ in. thick is added, and the whole boiled for an hour. Now put one teaspoonful of butter and one of flour into a small pan on the fire, add a teaspoonful of milk gradually, stirring all the time until well mixed; then add a teaspoonful of cream and mix with the lentil—or pea—pulp. To vary the flavour of the soup a sufficient quantity of green peas, spinach, asparagus, or other green vegetable, should be placed in cold water and boiled for half an hour, rubbed through a sieve, and added to the pulp. The soup should ultimately be more of the consistency of porridge than of ordinary soup.

Meat may be of any kind, but it is more easily digested if it is prepared as follows: It is cut up finely with a sharp knife, and thoroughly pounded in a mortar while raw. It is then mixed with sufficient beaten-up white of egg and milk to make a thick cream, placed in a china basin and boiled in a pan of water for three to five minutes, being well stirred during the process.

### Results of Treatment.

The result of the treatment is that after two or three days, during which there may be much painful flatulent distension of the abdomen, a natural action of the bowels occurs—the fæces being of a soft buttery consistence entirely different to the hard masses previously formed, and also different to the watery motions obtained by aperient medicines. When a daily evacuation has been really established the patient may be allowed to get up for two hours daily and go for a walk or drive, but it is usually necessary to continue the treatment for at least six weeks. After this a gradual return may be made to more ordinary diet; but plenty of coarse bread, coarse-fibred vegetables, and fat—the latter not necessarily undisguised—should remain constant constituents of the diet.

